# Cognitive dysfunction in Parkinson's disease: Hippocampal vulnerability and redox-driven mechanisms.

**DOI:** 10.1016/j.redox.2026.104318

**Published:** 2026-07-20

**Authors:** Ana Rita Curto, Ana Alexandra Silva, Mariana Bernardo Fiadeiro, Dina Pereira, Ana Clara Cristóvão

**Affiliations:** a RISE-Health, Department of Medical Sciences, Faculty of Health Science, University of Beira Interior, Av. Infante D.Henrique, Covilhã, 6200-506, Portugal; b NeuroSoV, UBIMedical, Municipal Road 506, Covilhã, 6200-284, Portugal; c CEG-IST, Center of Management Studies, Instituto Superior Técnico, University of Lisbon, Portugal; d University of Beira Interior, NECE, Research Center for Business Sciences and UBImedical, Portugal; e Polytechnic University of Castelo Branco, Higher School of Technology, Campus da Talagueira, Castelo Branco, Portugal

**Keywords:** Parkinson's disease, Cognitive impairment, Hippocampus, Oxidative stress, NADPH oxidase, Neuroinflammation

## Abstract

Cognitive dysfunction is one of the most disabling non-motor manifestations of Parkinson's disease (PD), progressing from mild cognitive impairment to Parkinson's disease dementia. Although multiple pathological processes have been individually implicated, the mechanisms linking neurotransmitter deficits, proteinopathies, circuit vulnerability, and neurodegeneration remain insufficiently integrated. Here, we synthesize current evidence on the pathophysiology of cognitive impairment in PD, emphasizing the convergence of dopaminergic, cholinergic, noradrenergic and serotonergic dysfunction with α-synuclein, tau and amyloid-β pathology. We highlight the hippocampus – particularly the CA2 subregion – as a critical anatomical hub connecting synaptic dysfunction, memory impairment, and dementia progression. Accumulating evidence identifies oxidative stress and neuroinflammation as central drivers across these pathological domains. Among endogenous sources of reactive oxygen species, NADPH oxidases (NOX), especially Nox4, emerge as key regulators of redox imbalance, protein aggregation and glial–neuronal interactions. Increased Nox4 activity correlates with hippocampal damage and cognitive decline, whereas experimental inhibition of Nox4 preserves synaptic integrity and improves memory performance in preclinical models. By integrating molecular, cellular and systems-level findings, this review positions redox dysregulation – and NOX-dependent signaling in particular – as a unifying mechanism underlying cognitive decline in PD, and discusses emerging therapeutic strategies targeting redox pathways, highlighting NOX modulation as a promising approach to modify the course of Parkinson's disease-associated cognitive impairment.

## Highlights

• NADPH oxidase 4 is a key driver of redox imbalance in Parkinson's disease.
• Nox4-derived ROS link oxidative stress to neuroinflammation and synaptic dysfunction.
• Redox dysregulation integrates protein aggregation with network-level dysfunction.
• The hippocampal CA2 subregion emerges as a vulnerable hub associated with cognitive decline.
• Targeting NOX-dependent pathways represents a potential strategy for modifying cognitive impairment in Parkinson's disease.

## Graphical abstract

**Redox mechanisms underlying cognitive dysfunction in PD.** Upregulation of NADPH oxidases (NOXs), specifically Nox4, drives reactive oxygen species (ROS) production, leading to severe oxidative stress. This pro-oxidant microenvironment drives pathological hallmarks: synaptic dysfunction, protein aggregation (formation of α-synuclein aggregates, amyloid-β plaques, and neurofibrillary tangles), and chronic neuroinflammation. These cellular alterations contribute significantly to hippocampal vulnerability, particularly focusing on the CA2 region, which serves as a critical neurological link accelerating the progression of cognitive dysfunction from early Parkinson's disease (Early PD) to Mild Cognitive Impairment (PD-MCI) and, ultimately, Parkinson's disease dementia (PDD). Created with BioRender.com.

**Figure:**
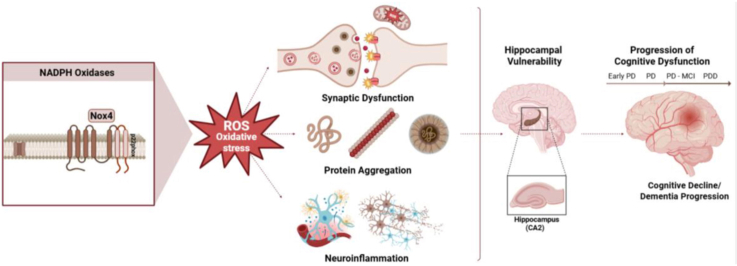

## 1 Introduction

### 1.1 Etiology and pathological hallmarks of Parkinson's disease

**Fig. 1:**
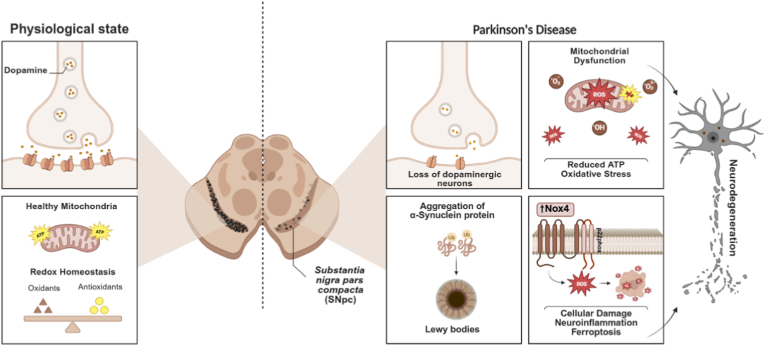
**Pathological features and molecular mechanisms of Parkinson's Disease (PD).** Under physiological conditions (left), which is characterized by normal dopaminergic neurotransmission and mitochondrial redox homeostasis, PD (right) exhibits a progressive loss of dopaminergic neurons in the *substantia nigra pars compacta* (SNpc) and a consequent decrease in dopamine levels. At the molecular level, the disease is driven by the aggregation of α-synuclein protein into Lewy bodies, mitochondrial dysfunction, characterized by reduced ATP production, increased reactive oxygen species (ROS), and the upregulation of Nox4. Together, these processes promote oxidative stress, cellular damage and, neuroinflammation, culminating in neurodegeneration. Adapted from “Progression of Parkinson's Disease in the *Substantia Nigra*”, with BioRender.com (2026). https://app.biorender.com/biorender-templates.

First described by James Parkinson in 1817, Parkinson's disease (PD) is the second most common neurodegenerative disease after Alzheimer's, with a global prevalence of more than six million individuals [1,2]. Its cause is still unknown for most diagnosed cases, and it has a multifactorial etiology, resulting from synergistic effects between environmental and genetic factors [3]. Pathologically, it is characterized by a progressive loss of dopaminergic neurons in the *substantia nigra pars compacta* (SNpc) located in the midbrain, which causes a decrease in the level of dopamine in the *striatum* and the formation of cytoplasmic inclusions, called Lewy bodies, containing insoluble α-synuclein aggregates (Fig. 1) [1,[4], [5], [6]]. However, PD is not limited to the loss of dopaminergic neurons of the SNpc, but also involves the dysfunction of cells, namely neurons [7] located in other regions of the neural network [[7], [8], [9]], including the dorsal motor nucleus of the *vagus* nerve [10], the *nucleus basalis* of Meynert [11], the medial/dorsal raphe [12], the *locus coeruleus* [11,12] and the pedunculopontine nucleus [8,13].

These neurodegenerative changes develop gradually and translate into a combination of motor and non-motor symptoms [6,14]. Motor symptoms include tremors, rigidity, bradykinesia (abnormally slow voluntary movements associated with cognitive impairment), and postural instability, resulting in balance problems [14]. On the other hand, non-motor symptoms involve a disturbed sense of smell, sleep problems, and constipation [14]. Some patients also have neuropsychiatric symptoms such as hallucinations, depression, anxiety, cognitive impairment, and dementia [6].

Over the years, various mechanisms have been described in the pathogenesis of PD, with the main one being the aggregation of α-synuclein [9]. However, several studies suggest that mitochondrial dysfunction [[15], [16], [17]], decreased proteasome function [18], neuroinflammation [19,20], and mutations in other genes play crucial roles in the onset and progression of the disease [9,21,22].

Oxidative stress (OS) is strongly associated with these cellular alterations, driven heavily by enzymatic systems such as nicotinamide adenine dinucleotide phosphate (NADPH) oxidases (NOX) [23]. These are multi-subunit enzyme complexes whose catalytic function is the production of reactive oxygen species (ROS), acting as key determinants of downstream neurodegeneration [[23], [24], [25], [26], [27]]. This oxidative stress causes the accumulation of harmful compounds that damage proteins, lipids, and enzymes, which, in the PD context, culminates in dopaminergic neuronal degeneration, being considered a key factor in the pathogenesis of the disease [28]. In this review, we specifically emphasize redox dysregulation – and NOX-dependent signaling, especially Nox4 – as a unifying mechanism that intersects dopaminergic, cholinergic, noradrenergic and serotonergic deficits with protein aggregation and hippocampal circuit vulnerability.

Among the mechanisms contributing to cognitive decline in PD, oxidative stress has emerged as a central pathological feature, with NOX, particularly Nox4, receiving increasing attention. NOX isoforms have been implicated in neuronal and glial processes that regulate both physiological redox signaling and pathological ROS overproduction, positioning Nox4 as a mechanistically relevant driver of Parkinson's Disease Dementia (PDD) [[29], [30], [31]]. Supporting this pathological role, clinical and experimental data have shown that Nox4 is significantly upregulated in patients with PD and is prominently expressed in the hippocampus [32], a key brain structure located in the temporal lobe that is responsible for learning and memory consolidation [33,34]. Experimental evidence demonstrates that Nox4 inhibition can reduce oxidative stress, attenuate neuroinflammation, and improve cognitive performance in animal models, highlighting the potential of NOX-targeted approaches to address domains that are unresponsive to dopaminergic therapies [35,36].

Reflecting the clinical urgency to target these progressive cognitive features, the study of cognitive impairment and dementia in PD has expanded substantially, reflecting the growing recognition of cognition as a major determinant of disease burden and quality of life [9,35]. Epidemiological data indicate that approximately 25-30% of individuals with PD meet criteria for mild cognitive impairment (MCI), and longitudinal cohorts show that up to 70% of these patients may progress to dementia within ten years of motor symptom onset [35]. Among the cortical–limbic structures affected, the hippocampus – particularly the CA2 subregion – emerges as a key anatomical hub in PD, integrating synaptic dysfunction, memory impairment, and the transition from mild cognitive impairment to dementia. This high conversion rate reinforces the need for a precise mechanistic understanding of the neuroanatomical and pathological hallmarks involved, particularly the impact of neurodegeneration on fronto-striatal and hippocampal circuits [29,37,38]. This cognitive deterioration in PD is driven by a complex interplay between neuroinflammation and the accumulation of pathological proteins, including α-synuclein, tau, and, in a subset of patients, amyloid-β [[39], [40], [41]]. However, large cohort studies highlight substantial heterogeneity in cognitive trajectories, showing that not all individuals with PD will develop dementia [36,41]. Understanding these divergent patterns is essential for improving prognostication and for designing targeted interventions that address distinct cognitive phenotypes [42,43]. Currently, therapeutic options for Parkinson's disease dementia remain limited to symptomatic strategies that do not alter disease progression [43,44]. Therefore, the growing body of literature underscores the urgent need for innovative, mechanistically grounded treatments, such as Nox4 inhibition, that may reshape the therapeutic landscape and improve clinical outcomes in the long term [36,44].

## 2 The clinical and neuroanatomical spectrum of cognitive decline in PD

**Fig. 2:**
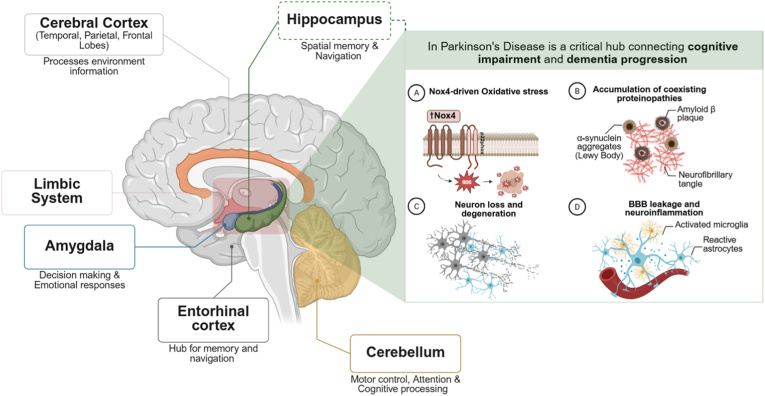
**Neuroanatomical regions in Parkinson's disease (PD) and the central role of the hippocampus in cognitive impairment.** Schematic representation of key brain structures and their physiological functions, including the cerebral cortex (environmental information processing), limbic system, amygdala (decision making and emotional responses), entorhinal cortex (memory hub), and *cerebellum* (motor control and cognitive processing). In PD, the hippocampus, normally responsible for spatial memory and navigation, acts as a critical hub connecting cognitive impairment and dementia progression. This pathological state is driven by distinct mechanisms: (A) Nox4-driven oxidative stress; (B/C) progressive neuron loss and degeneration; (C) accumulation of coexisting proteinopathies (including α-synuclein aggregates/Lewy bodies, amyloid-β plaques, and neurofibrillary tangles); and (D) blood-brain barrier (BBB) leakage accompanied by neuroinflammation involving activated microglia and reactive astrocytes. Adapted from “The Limbic System”, with BioRender.com (2026). Retrieved from https://app.biorender.com/biorender-templates.

Cognitive impairment in PD is complex, reflecting convergent neuropathological mechanisms that include diffuse protein aggregation, multi-system neurotransmitter loss, and selective damage to cortico-hippocampal circuits [41,45,46]. As summarized in Fig. 2, the hippocampus acts as a key hub for memory processing and spatial navigation, while its interaction with cortical, limbic, and subcortical networks helps explain the heterogeneity of cognitive symptoms observed across the disease spectrum [34,37,47,48]. Disruption of these interconnected networks contributes to deficits ranging from subtle processing abnormalities to clinically significant memory and executive dysfunction [46,49,50].

Clinically, cognitive impairment in PD is highly heterogeneous regarding symptom presentation, severity, and progression [46]. This clinical spectrum ranges from subtle oscillations such as bradyphrenia, which is characterized by slower processing, to mild cognitive impairment (MCI). The prevalence of MCI ranges significantly from 9 to 54%, likely due to differences in the criteria used [51]. On average, MCI appears in 15-25% of newly diagnosed patients and approximately 26% in non-recent cohorts [45,46,49]. MCI represents a pathological intermediate state where deficits are measurably greater than expected for typical aging, but not severe enough to impact the patient's daily functional independence. Although often associated with a deficit in a single domain, such as verbal fluency or memory loss, it increases the risk of converting to dementia up to sixfold [46,49]. Ultimately, this decline can progress to dementia, characterized by severe multi-domain deficits and overt functional impairment, which eventually develops in up to 80% of long-term patients, typically after 15 to 20 years [46,49,50].

### 2.1 Neurochemical imbalances and cross-disease pathophysiological correlates

The classic view of PD as a predominantly dopaminergic motor disorder has shifted toward the recognition of a broad spectrum of non-motor symptoms, many of which precede motor onset and become predominant as the disease progresses [[52], [53], [54], [55], [56]]. This evolution reflects the diffuse, preclinical propagation of pathological α-synuclein, encoded by *SNCA*, from presynaptic terminals to limbic and neocortical regions, where its accumulation drives cognitive decline and dementia [9,34,45,57]. In particular, atrophy of the entorhinal cortex and hippocampal subfields correlates with memory deficits and progression from PD-MCI to dementia, while higher cortical α-synuclein burden and coexisting amyloid-β and tau pathology exert additive, synergistic effects that accelerate cognitive deterioration and reduce survival [[58], [59], [60], [61], [62], [63]]. In longitudinal clinicopathological cohorts, PD patients with combined Lewy body, amyloid-β, and tau pathology show faster cognitive decline and shorter survival times than those with isolated Lewy body pathology [45,[58], [59], [60],^62^].

To explain these cognitive variations, the “dual syndrome” hypothesis clarifies differences in cognitive phenotype and progression [64]. First, the frontal-striatal network, modulated by dopamine, contributes to deficits in attention, working memory, planning, and response inhibition. In healthy individuals, cortical modulation of dopamine can increase working memory, attention, and visuospatial processing, and promote cognitive effort, suggesting a fundamental role for dopamine in cognitive function [45]. However, traditional dopamine replacement therapies provide symptomatic relief of motor symptoms but distribute dopamine non-selectively throughout the brain. As a result, while they can improve cognitive performance in areas with higher dopaminergic denervation, these treatments can simultaneously overload less affected regions, causing an “overdose” of dopamine, leading to localized impairments in dependent cognitive processes [[65], [66], [67]]. Second, the dual syndrome entails posterior cortical degeneration driven by cholinergic and noradrenergic loss, which accelerates progression toward dementia [46,64].

**Fig. 3:**
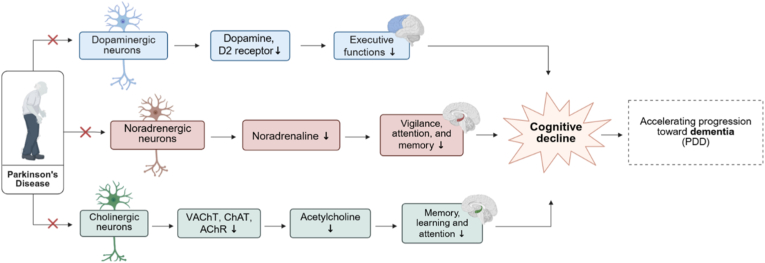
**Neurotransmitter pathways and cognitive symptom mapping in Parkinson's disease (PD).** The pathogenesis of PD involves the degeneration of multiple neurotransmitter systems that converge to drive cognitive impairment. The progressive loss of dopaminergic neurons is responsible for decreased levels of dopamine and its D2 receptors, impairing executive functions. Simultaneously, the loss of noradrenergic neurons reduces noradrenaline levels, leading to deficits in vigilance, attention, and memory. Furthermore, the degeneration of cholinergic neurons causes a decrease in critical cholinergic markers: vesicular acetylcholine transporter (VAChT), the enzyme choline acetyltransferase (ChAT), and the acetylcholine receptor (AChR), leading to reduced acetylcholine levels, resulting in impaired memory, learning, and attention. All these pathways culminate in cognitive decline, ultimately accelerating the progression toward Parkinson's disease dementia (PDD). Adapted from the article “Cognition Deficits in Parkinson's Disease: Mechanisms and Treatment” [68] and created with BioRender.com.

These neurochemical changes result from subcortical nuclei degeneration (Fig. 3) [66,68,69]. *Locus coeruleus* noradrenergic neurons [45] modulate alertness, arousal, attention, and memory. The frontal cortex and hippocampus receive dense noradrenergic innervation from this region [45], and reduced norepinephrine levels correlate strongly with cognitive decline in PD [45,70,71]. Concurrently, basal forebrain cholinergic neurons project to the neocortex, hippocampus, and amygdala [45,72]. Their loss reduces levels of vesicular acetylcholine transporter (VAChT), the enzyme choline acetyltransferase (ChAT), and the acetylcholine receptor (AChR), affecting memory, learning, and attention; notably, hippocampal acetylcholine receptor loss correlates with the severity of cognitive deficits [68]. Lastly, although brainstem serotonergic neurons degenerate pre-clinically, their loss directly associates with amyloid-β deposition, increasing the risk of cognitive decline [45,73,74]. Clinically, Olivola et al. [75] showed that PD patients had significantly reduced cerebrospinal fluid (CSF) levels of serotonin and its metabolite (5-HIAA) when compared to controls, though no direct correlation was found with motor or non-motor symptoms like depression [75], which over the decades has been correlated with serotonin impairment [76]. Mechanically, in APP/PS1 transgenic mice, an animal model of AD, and in cognitively normal elderly humans, selective serotonin reuptake inhibitors lowered amyloid load and plaque accumulation [73]. This suggests that serotonin signaling stimulates G-protein-coupled receptor cascades that favor α-secretase cleavage of amyloid precursor protein (APP), reducing toxic amyloid-Aβ peptide and reducing plaque formation [73,74].

Like PD, Huntington's disease (HD) primarily affects subcortical basal ganglia circuits and is characterized by early neuropsychiatric and cognitive changes, including depression, bradyphrenia, and executive dysfunction [77]. HD is a rare, progressive, autosomal dominant neurodegenerative disorder caused by the expansion of the cytosine-adenine-guanine (CAG) nucleotide triplet in the huntingtin gene, resulting in early and selective cell loss and atrophy of the caudate nucleus and putamen [77,78]. In both disorders, these symptoms may manifest during the presymptomatic phase, decades before the onset of motor symptoms [78]. Depression stands out as one of the most common psychological disorders in HD, affecting 33–69% of individuals with CAG expansions, with the highest incidence occurring immediately prior to clinical diagnosis. [79]. It is directly associated with accelerated functional and cognitive decline and a poorer quality of life [79]. Within the spectrum of these cognitive changes, bradyphrenia and executive deficits stand out as early manifestations in both conditions. [77]. However, as the diseases progress, their neuroanatomical trajectories diverge: PD involves progressive dysfunction of cortico-basal ganglia-thalamo-cortical loops, whereas HD shows early, selective degeneration of the caudate nucleus and putamen, with hippocampal involvement emerging later and remaining less well defined in human studies [77,[80], [81], [82]]. Taken together, these similarities emphasize that, despite overlapping clinical manifestations, hippocampal redox dysregulation appears to be considerably more central to cognitive decline in PD than in HD.

To evaluate these mechanisms, research has focused on the hippocampus, a complex bilateral seahorse-shaped structure within the medial temporal lobe, essential for memory learning and spatial navigation [34,48,83,84]. The hippocampus comprises the *dentate gyrus* (DG), where granule cells retain proliferative capacity to support adult neurogenesis [[85], [86], [87], [88]], the hippocampus itself (including the *Cornu Ammonis* (CA), which is divided into three sub-regions (CA1-CA3)), *subiculum*, *presubiculum*, *parasubiculum*, and the entorhinal cortex [34,48]. Pyramidal projection cells vary from large cells in the CA3 to smaller cells in CA1 [48]. Structural atrophy within this network predicts faster overall cognitive decline [47,89] and progression to MCI and dementia in PD [37,90], correlating with Lewy bodies and neurites density in the hippocampus [91] suggesting that the hippocampus plays an essential role in cognitive impairment, promoting progression to dementia in PD [29,[91], [92], [93], [94]]. Historically, the CA2 subregion received comparatively little attention in PD research, even though early neuropathological studies had already demonstrated a high burden of Lewy neurites and a tight correlation with cognitive decline in PD patients [92,94,95]. More recent functional, imaging, and translational work has firmly consolidated CA2 as a critical hippocampal hub linking synaptic dysfunction, memory impairment, and progression to dementia [34,48,96,97].

These pathological interactions are further corroborated by translational animal models. Transgenic (Thy1)-h[A30P]αSYN mice express mutated, fibril-forming α-synuclein and show widespread pathology across memory-critical brain regions that positively correlates with cognitive decline in Morris Water Maze and avoidance tests, effectively reproducing PDD phenotypes [98]. Combining deposition of α-syn and amyloid-β exacerbates fibril formation and accelerates cognitive decline [98,99].

To model sporadic PD, pioneering studies in non-human primates and rodents used the neurotoxin 1-methyl-4-phenyl-1,2,3,6-tetrahydropyridine (MPTP), which crosses the blood-brain barrier and is converted to the toxic metabolite MPP^+^. MPP^+^ is selectively taken up by dopaminergic neurons via the dopamine transporter and acts as a potent inhibitor of mitochondrial complex I, reducing ATP synthesis and triggering ROS production that induces neurotoxicity in the substantia nigra [5,[100], [101], [102]]. Mechanistically, these deficits reflect a simultaneous disruption of complex frontostriatal circuits, driven by severe dopamine depletion in the caudate nucleus and a reduction of norepinephrine in the frontal cortex, reproducing neocortical dysfunctions similar to those reported in human PD patients [98]. Similarly, MPTP-treated rodents show significant impairments in learning and long-term memory during the Morris Water Maze [103], indicating that severe dopamine deficiency degeneration reduces dopamine levels not only in the *striatum* but also compromises hippocampus-dependent cognitive functions [98,104].

## 3 Mechanistic role of oxidative stress in hippocampal dysfunction

### 3.1 Oxidative stress and free radicals dynamics

The term oxidative stress (OS) describes a redox imbalance between the production of free radicals or other reactive species and antioxidant defenses, and can be related to alterations in microglia activation, protein elimination, mitochondrial function, and the autophagy-lysosome system [23]. The central nervous system (CNS) is especially vulnerable to oxidative insult due to its exceptionally high oxygen consumption, limited antioxidant defenses, and a high concentration of easily oxidized polyunsaturated fatty acids (PUFAs) [27,105,106]. The CNS requires substantial energy production by mitochondria due to its high metabolic rate, which is necessary to generate ATP via the electron transport chain and oxidative phosphorylation. This high metabolic activity means that neurons and glia - microglia and astrocytes - are more susceptible to producing large quantities of reactive oxygen species (ROS) and reactive nitrogen species (RNS) [[107], [108], [109]].

Notably, this oxidative vulnerability is particularly pronounced in specific neuronal populations, such as nigrostriatal dopaminergic neurons [[110], [111], [112]]. This energetic demand is especially prominent in substantia nigra dopaminergic neurons, whose autonomous pacemaker firing relies on continuous Ca^2+^ influx and tight mitochondrial buffering, creating a chronic metabolic and oxidative burden. These cells exhibit autonomous pacemaker activity that depends on a continuous influx of Ca^2+^, requiring rigorous regulation of CaMKIIα–Ca^2+^/calmodulin signaling and efficient mitochondrial Ca^2+^ buffering [110,113]. Although mitochondria help control cytosolic Ca^2+^ levels, this process is intrinsically linked to ROS generation. Consequently, these neurons maintain inherently elevated basal levels of both Ca^2+^ and ROS, which impose a chronic metabolic and oxidative burden [110,112]. This microenvironment is further aggravated by cell-specific factors, including a high neuromelanin content and the production and auto-oxidation of dopamine, which generates toxic quinones capable of generating additional ROS and inducing structural and conformational changes that promote incorrect folding and protein aggregation [23,[114], [115], [116]]. This limited buffering capacity ultimately explains their heightened susceptibility to selective nigrostriatal damage upon systemic exposure to environmental mitochondrial toxins, such as pesticides [110,113]

The best-known ROS and RNS include singlet oxygen (O_2_), superoxide anion (O_2_^•−)^, hydroxyl radical (HO^•^), hydrogen peroxide (H_2_O_2)_, nitric oxide (NO), and peroxynitrite anion (ONOO^−^) [105,109]. Within the CNS, the two main sources of endogenous ROS are the mitochondria and the NADPH oxidase (NOX) pathway [107,117]. Mitochondria are the main intracellular source of ROS, producing them as byproducts of aerobic respiration mediated by the electron transporter chain. NOX catalyzes the transfer of electrons from NADPH to oxygen, which is eventually converted into ROS [109,117]. Additionally, ROS can also be produced by other oxidases, namely cytochrome P450, xanthine oxidases, and lipoxygenases, among others [107,111,118].

Under physiological conditions, basal levels of ROS and RNS play an important role in various body functions, including cell signaling, host defense, gene expression regulation, and programmed cell death [23,28,107]. To maintain homeostasis and prevent damage, the antioxidant system works together with small amounts of ROS, involving a balance between pro-oxidant substances and antioxidant substances. Antioxidants are a group of protective factors that include enzymes such as superoxide dismutase (SOD), catalase (CAT), glutathione peroxidase (GPx), and glutathione-S-transferase, and non-enzymatic factors such as melatonin, which interact with each other to protect the body against damage caused by ROS [28,107]. Within this network, SOD operates through distinct isoenzymes, including cytosolic SOD1 and mitochondrial SOD2, which catalyze the conversion of superoxide into H_2_O_2_ and molecular oxygen; subsequently, the H_2_O_2_ is degraded by GPx and CAT [114,119]. This enzymatic defense is closely coupled with the glutathione system, where reduced glutathione (GSH) is synthesized from the amino acid cysteine, which acts as the rate-limiting factor in its synthesis [114,119]. GSH interacts directly with ROS and reduces peroxynitrite, hydroxyl, and superoxide radicals, making the reduced-to-oxidized glutathione ratio (GSH/GSSG) an accurate reflection of cellular redox homeostasis [114,120]. Notably, disturbances in NADPH levels, an essential cofactor for the enzyme glutathione reductase, can severely affect this ratio. It is concluded that in PD, levels of both GSH and NADPH, which are necessary for the reduction and recycling of GSSG back to GSH, are significantly decreased, resulting in a marked decline in the GSH/GSSG ratio [120,121]

Crucially, the nuclear factor erythroid 2-related factor 2 (Nrf2) is the master transcription factor governing the endogenous antioxidant system, controlling its expression and maintaining redox homeostasis in the body [122,123]. In fact, Nrf2 precisely regulates the balance between the reduced and oxidized forms of glutathione [124]. Under normal conditions, Nrf2 is sequestered in the cytoplasm by KEAP1 (Kelch-like ECH-associated protein 1), targeting it for ubiquitin-mediated proteasomal degradation. KEAP1 contains highly reactive cysteine residues that serve as functional stress sensors; in response to OS, these cysteines undergo modifications, such as S-alkylation, S-nitrosylation, S-glutathionylation, and S-sulfhydrylation [123]. The resulting conformational changes stabilize Nrf2, allowing it to escape proteasomal degradation and translocate into the nucleus. Here, it binds to antioxidant response elements (ARE) located in the promoter regions of target genes to promote the transcription of antioxidant enzymes [122,123]. The development of PD is directly correlated with a dysfunction of this pathway [125]. Interestingly, while Nrf2 is normally localized in the cytosol under physiological conditions, it is highly present in the nucleus of PD patients, indicating the activation of an OS response in the brain [122,125]. However, sustained activation of NOX enzymes, particularly Nox4, is frequently accompanied by the functional inhibition of Nrf2 signaling [126]. Consequently, this Nrf2 deficiency is directly linked to the progression of PD [122].

When this critical pathway is overwhelmed and mitochondrial quality control mechanisms are compromised, a runaway production of ROS rapidly overloads the cell's remaining baseline antioxidant capacity [123]. This persistent redox imbalance drives the accumulation of harmful compounds that damage proteins, enzymes, and lipids, ultimately compromising dopaminergic neurons' viability. This destructive cascade downregulates key antioxidant enzymes, such as GPx and SOD, resulting in an excessive increase in ROS levels and the consequent depletion of antioxidant reserves [28]. Direct clinical evidence of this systemic collapse is reflected in studies of *post-mortem* brain tissue, which exhibit an increase in the oxidation of proteins [127], lipids [128,129], and DNA [130,131] as well as a decrease in the levels of the antioxidant molecule, glutathione, in the SN of PD patients [[132], [133], [134]]. This localized vulnerability is amplified by the fact that SN possesses the highest concentration of microglia cells in humans and rodents, whose chronic activation can lead to an excessive level of oxidative stress [23]. Consistently, both *post-mortem* PD brains and experimental models of the disease demonstrate elevated levels of oxidative stress markers, including 8-hydroxy-2′-deoxyguanosine (8-OHdG) [127,[135], [136], [137]], 4-hydroxy-2-nonenal (HNE) [[138], [139], [140]], and malondialdehyde (MDA) [141].

Since α-synuclein aggregates are the main pathological hallmark of the disease, their possible relationship with OS has been studied. Several studies, *in vitro* and *in vivo*, have shown that ROS and, subsequently, OS promote the formation of α-synuclein aggregates [[142], [143], [144], [145], [146]]. Indeed, inhibition of mitochondrial complex I through exposure to toxins such as MPTP or rotenone results in aggregation and the formation of α-synuclein-positive intraneuronal inclusions in dopaminergic neurons [147,148], suggesting that mitochondrial dysfunction could result in aggregation of α-synuclein [149]. In turn, the unfolding of α-synuclein into toxic oligomers induces the production of ROS, establishing a bidirectional relationship [143,145]. Pathological α-synuclein interacts with the mitochondrial import machinery by binding to the TOM20 receptor, which impedes protein import and results in the accumulation of dysfunctional mitochondria. Additionally, oligomeric forms enter or directly associate with mitochondria, where they interact with complex I and ATP synthase [145,149,150]. This interaction impairs cellular respiration, reduces the efficiency of ATP synthesis, and causes electron leakage that increases ROS production. α-Synuclein also affects mitochondrial dynamics by inhibiting fusion and increasing fission, thereby compromising mitochondrial integrity [149]. In fact, seeding with preformed α-synuclein fibrils (PFFs) causes a reduction in the expression of a wide range of somatic genes that encode oxidative phosphorylation proteins, resulting in a functional collapse in which mitochondria cease to produce energy and begin to import ATP from the cytosol to maintain their polarization, thereby leading to disrupted mitochondrial function [151]. In parallel, α-synuclein PFF seeding induced a down-regulation in the expression of genes related to dopamine synthesis, sequestration and, release [151]. Once the aggregation is initiated, even small concentrations of toxic oligomers are sufficient to induce marked ROS production, exacerbate OS, and cause neuronal death [143]. In this context, redox-active metals such as iron and copper, which are generators of ROS, have been found to accumulate in the brains of PD patients [152]. Under physiological conditions, redox-active metals like copper or iron are sequestered, maintaining minimal free concentrations [153]. However, in neurodegenerative diseases, elevated levels of free metals can react with H_2_O_2_ via the Fenton reaction, resulting in the formation of highly damaging hydroxyl radicals [107,153,154]. Additionally, free iron catalyzes the Haber–Weiss reaction, combining hydrogen peroxide and superoxide anions to generate further HO^•^. Due to their powerful oxidation potential, these hydroxyl radicals are among the most reactive species in biological systems, aggressively oxidizing a wide range of organic molecules [[153], [154], [155]]. Crucially, when present in excess, these ROS attack and react with PUFAs, leading to lipid peroxidation [121,156]. These oxidized products can also react with adjacent molecules, triggering a chain reaction that damages the entire membrane structure and ultimately promotes ferroptosis, a regulated form of iron-dependent cell death. The excessive accumulation of these lipid peroxides, converted from PUFAs, is the primary driver of this process, fundamentally impairing the integrity of the plasma membrane lipid bilayer and accelerating cellular collapse [121,156]

Beyond direct neuronal death, this oxidative environment affects other vital cellular components of the CNS [109,141], such as astrocytes [109]. Astrocytes, which are the main cells maintaining CNS homeostasis, nutrient supply, and BBB integrity, are deeply impacted [117,157]. OS induces astrocytic inflammation and reactive astrogliosis, a process mediated by ROS/RNS, which activate pro-inflammatory signaling pathways and promote the release of cytokines [117]. Specifically, ROS can activate the NLRP3 inflammasome, activating procaspase-1 into active caspase-1, which promotes the release of pro-inflammatory cytokines, thereby exacerbating neuronal damage [117]. Because astrocytes and microglia are in constant communication, these pro-inflammatory cytokines released by astrocytes activate microglia [109]. Since the SN contains the highest density of microglial cells, their subsequent over-activation generates an excessive level of OS, reinforcing astrogliosis and promoting a vicious cycle that contributes to the progression of neurodegeneration [23,109]

As the disease progresses, this neurodegeneration spreads beyond the nigrostriatal system to other brain regions, including the hippocampus, amygdala, thalamus, cerebellum, and prefrontal cortex [23,109,115,141]. The hippocampus, amygdala, and prefrontal cortex stand out as highly vulnerable targets due to their particularly high energy requirements, intrinsic differences in DNA repair capacity, and changes in calcium-dependent signaling mechanisms [109]. Consequently, these structures show functional changes earliest under conditions of OS [23,109,115].

This extensive oxidative damage and atrophy lead to the onset of clinical symptoms that go beyond the typical motor features of PD, driving non-motor symptoms such as cognitive deficits, memory impairment, learning difficulties, anxiety, and depression [141,158]. This theory is strongly corroborated by *in vivo* experimental data showing that chronic stress and prolonged ROS exposure cause a significant reduction in cortical and hippocampal antioxidant enzymes alongside increased lipid peroxidation and MDA production, which positively correlate with the severity of cognitive impairment and depression in animal models of the disease [141,[158], [159], [160]]

Consequently, comprehensive neuropsychological assessments based on the Movement Disorder Society criteria remain vital to identify progression to dementia, a trajectory compounded by factors that exacerbate underlying oxidative stress and neuroinflammation [45,46,[161], [162], [163]]. Ultimately, these findings confirm that ROS overproduction may contribute to damage in various brain regions, explaining the emergence of cognitive phenotypes independent of classic dopamine deficiency [141].

### 3.2 NADPH oxidases isoforms as drivers of cortico-hippocampal dementia

The enzymes nicotinamide adenine dinucleotide phosphate oxidases (NADPH oxidases - NOX), which produce reactive oxygen species as their primary function, are considered the main enzymatic source of oxidative stress in PD [23]. NOX are multi-subunit enzyme complexes whose catalytic function is the production of ROS. They function as membrane electron transporters, using reduced NADPH as an electron donor and molecular oxygen as an electron acceptor, which results in the production of the superoxide anion and, subsequently, ROS, including hydrogen peroxide and hydroxyl radicals. To date, the NOX family consists of 7 members, which combine with various subunits to form active enzyme complexes - Nox1, Nox2, Nox3, Nox4, Nox5, dual oxidase 1 and 2 (Duox1/2) [27,106,126]. The NOX and DUOXisoforms are structurally similar, with each isoform presenting at least six α-helix transmembrane domains, binding sites for heme, flavin adenine dinucleotide (FAD), and NADPH [164]. Most of these isoforms require a complex process to become active; for example, Nox1, Nox2, and Nox3 depend on the stabilizing protein p22phox and the recruitment of specific cytosolic regulatory subunits, while Nox5 and Duox1/2 are activated by binding to intracellular calcium. In contrast, Nox4 exhibits distinct structural and regulatory characteristics; although it requires interaction with p22phox to ensure its stability, its activation does not depend on the assembly of cytosolic subunits. Instead, Nox4 exhibits constitutive catalytic activity, continuously generating ROS in the absence of specific external stimuli [27,126].

Given their importance in the development and progression of PD, NOX have been the subject of several studies, and it has been shown that neurons express Nox1 and Nox4, with Nox4 being a constitutively active isoform associated with various inner membranes, including the mitochondrial membrane [23,165,166]. In *post-mortem* patients, Nox1 and Nox4 have been found in the nucleus of dopaminergic neurons [23]. Nox4's downstream effects are believed to be mediated by hydrogen peroxide derived from superoxide. A variety of inflammatory and neurodegenerative factors, including APP, tumor necrosis factor alpha (TNF-α), interleukins, and α-synuclein, as well as neuronal damage and cell death, can increase NOX activation [164]. In the brains of *post-mortem* PD patients, signs of oxidative damage have been observed in degenerated areas, such as the SN [167] It has been demonstrated and supported by the increased activation of glial cells, appearing to be a persistent chronic activation, especially of microglia, which produce ROS through NOX, causing damage to dopaminergic neurons [164].

**Fig. 4:**
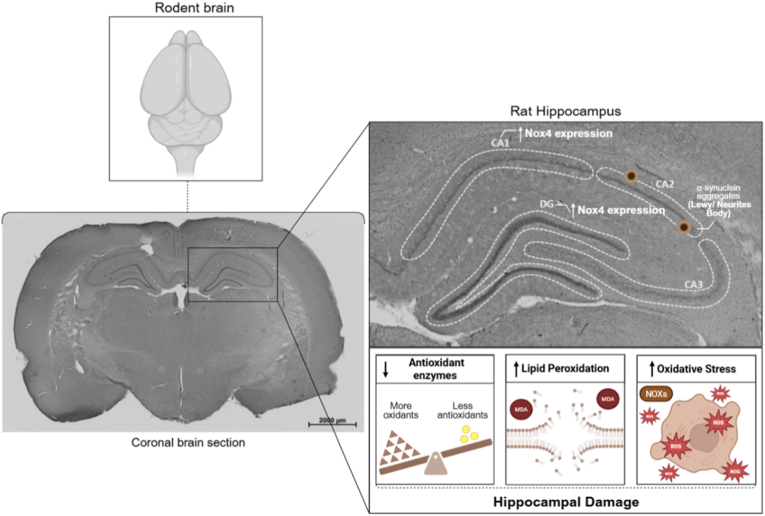
**Pathological alterations and oxidative stress mechanisms in the rat hippocampal formation.** Representative image of a coronal brain section captured using an AxioImager Z2 optical microscope with 5x objective (Stereology, ZEISS). Under pathological conditions, regional vulnerability is highlighted by increased Nox4 expression in the CA1 and DG areas, alongside the accumulation of α-synuclein aggregates (Lewy bodies and Lewy neurites) within the CA2 region. These alterations drive overall hippocampal damage through three convergent mechanisms: a decrease in antioxidant enzymes leading to a pro-oxidant imbalance, increased lipid peroxidation, evidenced by MDA accumulation and membrane damage, and elevated oxidative stress mediated by NOXs/ROS accumulation. Scale bar = 2000 μm. Created with BioRender.com.

Since Nox4 has been found in the brains of PD patients and is present in all neurons, astrocytes, and microglia, it has been suggested that NOX-derived ROS, particularly Nox1 and Nox4, are associated with the progression of PD [[24], [25], [26], [27],^168^,^169^]. A study by Hou et al. demonstrated that inhibiting NOX by apocynin in an animal model of PD improved neurodegeneration, synaptic loss, α-synuclein expression, and Ser129 phosphorylation in the hippocampus and/or cortex induced by neurotoxins by suppressing oxidative stress and neuroinflammation. Importantly, the specific NOX isoforms responsible for these effects could not be isolated, since apocynin acts as a broad-spectrum, non-selective NOX inhibitor. [126,168], Despite these pharmacological limitations, the direct role of specific isoforms is supported by targeted studies; for instance, the silencing of Nox4 in dopaminergic neurons, inducing a reduction in neurotoxicity and prevention of cognitive decline have been observed, suggesting a direct role for neuronal Nox4 in PDD [170]. In addition, Nox4 is present in astrocytes, and elevated levels of ROS during stressful conditions can contribute to astrocyte dysfunction, causing neurotoxicity. On the other hand, Nox4 is upregulated in *post-mortem* PD patients and is highly expressed in the hippocampus (Fig. 4), where it has a critical impact on dementia symptoms [32]. Consistent data indicate that Nox4 directly exacerbates the pathology of Parkinson's disease by promoting neuronal ferroptosis and neuroinflammation [32,166]. Specifically, it has been shown that pharmacological suppression of Nox4 increases dopaminergic GSH levels and reduces lipid peroxidation, thereby alleviating dopaminergic neuronal death in MPTP-induced PD models [166]. Similarly, inactivation of Nox4 effectively attenuates lipid peroxidation in astrocytes in the hippocampus under identical neurotoxic conditions [32]. In addition to promoting direct oxidative damage, Nox4 further accelerates cell death by suppressing the endogenous Nrf2/GPX4 antioxidant defense pathway [126].

Studies in transgenic mice have also reported that age-related cognitive impairment is linked to an increase in NOX activity, which leads to oxidative stress and amyloid-β deposition caused by increased expression of Nox2 and Nox4 [[171], [172], [173], [174]]. To investigate the role of NOX in the pathology of α-synuclein, tau protein and amyloid-β in the hippocampus, which is associated with PD with dementia, Nox4 expression was analyzed in an animal model, showing that the expression of Nox4 is positively regulated in the hippocampus [29,32,165,170], especially in the dentate gyrus, and that it is colocalized with the expression of the A11 oligomer [29]. These results suggest that cognitive impairment in PD with dementia could be caused by an increase in Nox4 activity in the hippocampus [29,165], which induces the expression of amyloid-β and the production of A11 oligomers. However, the exact mechanism by which Nox4 is involved in the synthesis and production of amyloid-β remains unknown [29].

### 3.3 Targeting NOX pathways: emerging therapeutic strategies

Since NOX contributes to the development and progression of PD, modulating its activity represents a potential therapeutic target capable of modifying the natural course of the disease [23,27,165,168]. In recent years, several chemical compounds that inhibit NOX were investigated; however, severe translational obstacles, such as limited specificity, selectivity, and potential toxicity, make them unsuitable for human use [175]. Furthermore, it is necessary to consider the potential impact that inhibitors may have on the physiological functions of NOX in healthy tissues, since excessive inhibition of NOX not only removes its basal and necessary function for normal cellular functioning but could also contribute to an increased risk of infections and various types of disease [23,165].

To overcome these structural limitations, considerable efforts have been focused on the development of highly selective, next-generation NOX inhibitors. The pyrazolopyridine dione derivatives, such as GKT137831 (Setanaxib) and GKT136901, represent a crucial advancement as dual Nox1/4 inhibitors, exhibiting highly promising pharmacological properties [126]. These dual Nox1/4 inhibitors represent a major advance in isoform-selective NOX pharmacology, exhibiting favorable pharmacokinetic properties and potent suppression of Nox1/4-mediated ROS production. Crucially, several of these selective Nox1/4 inhibitors have already entered clinical trials for systemic conditions, including fibrotic and inflammatory diseases, where they have been shown to be safe and well tolerated in humans, thereby validating their translational safety profile [[176], [177], [178], [179], [180]]

In CNS models, GKT137831 effectively mitigates PD-associated features by reducing oxidative stress, while preserving the integrity of the BBB. Similarly, its structural analog, GKT136901, acts as an effective dual inhibitor that reduces neuronal death in the hippocampus [181] and suppresses hypoxic endothelial permeability by specifically blocking Nox4-mediated hydrogen peroxide production [178,182].

By further refining this isoform-specificity, the GLX series of inhibitors represents a new generation of molecules, designed with exceptional selectivity for the Nox4 isoform, in order to completely minimize off-target effects on other NOX enzymes [126]. Within this group, GLX351322 significantly reduces ROS production, restores mitochondrial membrane potential, and rescues mitochondrial complex activity, effectively reversing neuronal apoptosis induced by mitochondrial dysfunction in PD-related cellular models [183].

In parallel models of Alzheimer's disease, the utilization of a Nox4 inhibitor, GLX351322, demonstrated its potential to protect against synaptic and memory dysfunction [184] in APP/PS1 transgenic mice, while reducing levels of amyloid-β and oxidative stress in the mice's hippocampus, highlighting the ability of Nox4 inhibition to reverse oxidative stress in the hippocampus, prevent neuronal death induced by amyloid-β peptide (Aβ_1–42),_ and improve synaptic and memory deficits [184,185].

Notably, it has been reported that both GLX351322 and its nanoformulations suppress neuronal ferroptosis in the hippocampus, one of the main mechanisms underlying nigral neuronal loss in PD, through downregulation of Nox4 expression, the subsequent activation of the protective Nrf2/HO-1/GPX4 antioxidant pathway, and reduction of both lipid peroxidation and iron accumulation [186].

Overall, these selective chemical advances suggest that, while broad-spectrum inhibition remains risky, targeted modulation of Nox4 and Nox1/4 holds immense multi-target therapeutic value for preserving the neurovascular unit and preventing mitochondrial dysfunction during the progression of PD. Ultimately, further specialized clinical trials and comprehensive evaluations are needed to thoroughly explore the exact role of these selective inhibitors in the pathophysiology of neurodegenerative conditions, particularly PD with dementia, given its increasing cumulative prevalence [68,126,165].

## 4 Conclusion

Cognitive impairment and dementia in PD present profound challenges, accelerating morbidity and mortality while affecting patients, caregivers, and healthcare systems. The progression from mild cognitive impairment to dementia in PD not only impairs cognitive functions but also exacerbates motor and non-motor symptoms, leading to a profound decline in overall quality of life. Current pharmacological treatments, such as cholinesterase inhibitors, offer only temporary, partial symptomatic relief and fail to modify the natural course of the disease, whereas standard l-DOPA therapies remain largely ineffective against these cognitive symptoms. This clinical gap underscores the urgent need for a deeper understanding of alternative pathological mechanisms and the development of disease-modifying therapies. In this context, targeting redox dysregulation via NOX modulation represents a highly promising therapeutic approach. Accumulating evidence indicates that overactivation of NOX isoforms, particularly Nox1 and Nox4, drives hippocampal oxidative stress, lipid peroxidation, and neuroinflammation, thereby compromising cortico–hippocampal circuits and accelerating the transition from PD-MCI to dementia. While early broad-spectrum NOX inhibitors faced major translational barriers due to limited isoform selectivity, potential toxicity, and disruption of essential physiological ROS signaling, the emergence of next-generation isoform-selective compounds, including dual Nox1/4 inhibitors and highly selective GLX derivatives, opens a strategic window for clinical translation. By preserving redox homeostasis, synaptic integrity, and cognitive function in preclinical models, selective Nox4 modulation represents one of the most promising disease-modifying therapeutic strategies for Parkinson's disease-associated cognitive impairment.

Collectively, current evidence identifies Nox4-dependent redox dysregulation as a mechanistic hub linking oxidative stress, protein aggregation, neuroinflammation and hippocampal dysfunction. Consequently, selective Nox4 modulation represents one of the most promising disease-modifying therapeutic strategies to slow disease progression and preserve cognitive function in Parkinson's disease-associated cognitive impairment. Nevertheless, further investigation into the structural biology and precise regulatory mechanisms of Nox4 will be essential to accelerate successful clinical translation.

## Author disclosure statement

The authors declare the following financial interests/personal relationships which may be considered as potential competing interests: Ana Clara Cristovao and Dina Pereira reports a relationship with FastPrinciple-Lda that includes board membership and equity or stocks. Ana Clara Cristovao and Dina Pereira have a patent that was licensed to FastPrinciple-Lda.

## Declaration of generative AI and AI-assisted technologies in the manuscript preparation process

During the preparation of this work, the authors used ChatGPT 5.2 (OpenAI, 2025) to copyedit and improve the clarity of the text. After using this tool/service, the authors reviewed and edited the content as needed and take full responsibility for the content of the published article.

## Funding information

This work was supported by the project CogniPD + Reference: CENTRO2030-FEDER-02960700/24252, financed by PT2030 and 10.13039/100006826EU Fundos Europeus Estruturais e de Investimento. Ana R. Curto is financially supported by 10.13039/501100001871FCT fellowship ref: 2024.00608.BDANA. Mariana B. Fiadeiro was financially supported by 10.13039/501100001871FCT fellowship ref: 2021.05277.10.13039/100017412BD. Ana A. Silva is financially supported by 10.13039/501100001871FCT fellowship ref: 2023.01129.BDANA.

## CRediT authorship contribution statement

**Ana Rita Curto:** Conceptualization, Investigation, Writing – original draft, Writing – review & editing. **Ana Alexandra Silva:** Writing – review & editing. **Mariana Bernardo Fiadeiro:** Supervision, Writing – review & editing. **Dina Pereira:** Conceptualization, Funding acquisition, Supervision, Writing – review & editing. **Ana Clara Cristóvão:** Conceptualization, Formal analysis, Funding acquisition, Investigation, Project administration, Resources, Supervision, Validation, Writing – review & editing.

## Declaration of competing interest

The authors declare the following financial interests/personal relationships which may be considered as potential competing interests: Ana Clara Cristovao reports financial support was provided by PT2030 and EU Fundos Europeus Estruturais e de Investimento. Ana Rita Curto reports financial support was provided by Fundação para a Ciência e Tecnologia (FCT). Ana Alexandra Silva reports financial support was provided by Fundação para a Ciência e Tecnologia (FCT). Mariana Fiadeiro reports financial support was provided by Fundação para a Ciência e Tecnologia (FCT). Ana Clara Cristovao reports a relationship with Fastprinciple Lda that includes: board membership and equity or stocks. Dina Pereira reports a relationship with Fastprinciple Lda that includes: board membership and equity or stocks. If there are other authors, they declare that they have no known competing financial interests or personal relationships that could have appeared to influence the work reported in this paper.

## Data Availability

No data was used for the research described in the article.

## 7. Glossary:

**4-HNE (4-Hydroxy-2-nonenal):** A highly reactive cytotoxic product of lipid peroxidation used as a biomarker for oxidative damage.

**8-OHdG (8-Hydroxy-2′-deoxyguanosine):** An oxidized derivative of deoxyguanosine, serving as a biomarker for oxidative DNA damage.

**Amyloid-β**: Peptide that forms extracellular plaques and may coexist with α-synuclein pathology in Parkinson's disease.

**ARE (Antioxidant Response Element):** Promoter DNA sequence that binds Nrf2 to initiate the transcription of antioxidant genes.

**ATP (Adenosine Triphosphate):** The primary energy source, whose mitochondrial synthesis is suppressed by complex I inhibitors like MPP+.

**Bradyphrenia:** Slowing of cognitive processing commonly observed in Parkinson's disease.

**CA2 (Cornu Ammonis 2):** A hippocampal subregion selectively affected in Parkinson's disease and associated with cognitive dysfunction.

**CaMKIIα–Ca^2+^/calmodulin (Ca^2+^/calmodulin-depedent protein kinase II):** A calcium/calmodulin-dependent protein kinase integral to regulating synaptic plasticity, continuous calcium influx, and mitochondrial buffering in pacemaker neurons.

**Cognition:** Mental processes involved in acquiring, processing, and using information, including attention, memory, language, and executive functions.

**Dopaminergic neurons:** Dopamine-producing neurons are primarily located in the substantia nigra pars compacta.

**GSH/GSSG:** Reduced glutathione (GSH), the primary endogenous non-enzymatic antioxidant, and oxidized glutathione disulfide (GSSG); their structural ratio reflects intracellular redox homeostasis.

**Hippocampus:** A medial temporal lobe structure essential for memory formation and spatial processing.

**Lewy bodies:** Intracellular inclusions composed mainly of aggregated α-synuclein.

**MDA (Malondialdehyde):** A highly reactive byproduct of lipid peroxidation that serves as a biomarker for oxidative stress and cell membrane damage.

**NOX (NADPH oxidases):** A family of enzyme complexes that generate reactive oxygen species through electron transfer from NADPH to molecular oxygen.

**Nrf2 (Nuclear factor erythroid 2-related factor 2):** The master transcription factor that coordinates the cellular antioxidant defense mechanism by translocating into the nucleus during oxidative conditions to bind ARE regions.

**Oxidative stress:** Imbalance between reactive oxygen species production and antioxidant defenses.

**PDD (Parkinson's disease dementia):** Dementia syndrome occurring in the context of established Parkinson's disease.

**PD-MCI (Parkinson's disease mild cognitive impairment):** Cognitive impairment in Parkinson's disease not severe enough to meet criteria for dementia.

**ROS (Reactive oxygen species):** Chemically reactive oxygen-containing molecules generated during metabolism and enzymatic reactions.

**SNpc (Substantia nigra pars compacta):** Midbrain region containing dopaminergic neurons that degenerate in Parkinson's disease.

**Tau protein:** Microtubule-associated protein that forms neurofibrillary tangles in neurodegenerative disorders.

**α-Synuclein:** Presynaptic protein encoded by *SNCA* that aggregates into insoluble fibrils in Parkinson's disease.

## References

[1] Simon D.K., Tanner C.M., Brundin P. (2020). Parkinson disease epidemiology, pathology, genetics, and pathophysiology. Clin. Geriatr. Med..

[2] Tolosa E., Garrido A., Scholz S.W., Poewe W. (2021). Challenges in the diagnosis of parkinson's disease. Lancet Neurol..

[3] Cerri S., Mus L., Blandini F. (2019). Parkinson's disease in women and men: what's the difference?. J. Parkinsons Dis..

[4] Balestrino R., Schapira A.H.V. (2020). Parkinson disease. Eur. J. Neurol..

[5] Chia S.J., Tan E.K., Chao Y.X. (2020). Historical perspective: models of parkinson's disease. Int. J. Mol. Sci..

[6] Navaratnam P., Arcona S., Friedman H.S., Leoni M., Shaik S., Sasane R. (2022). Natural history and patterns of treatment change in Parkinson's disease: a retrospective chart review. Clin Park Relat Disord.

[7] Kamath T., Macosko E.Z. (2023). Insights into neurodegeneration in Parkinson's disease from single-cell and spatial genomics. Mov. Disord..

[8] Giguere N., Burke Nanni S., Trudeau L.E. (2018). On cell loss and selective vulnerability of neuronal populations in parkinson's Disease. Front. Neurol..

[9] Kouli A., Torsney K.M., Kuan W.L., Stoker T.B., Greenland J.C. (2018). Parkinson's disease: Pathogenesis and Clinical Aspects.

[10] Halliday G.M., Blumbergs P.C., Cotton R.G., Blessing W.W., Geffen L.B. (1990). Loss of brainstem serotonin- and substance P-containing neurons in Parkinson's disease. Brain Res..

[11] Zarow C., Lyness S.A., Mortimer J.A., Chui H.C. (2003). Neuronal loss is greater in the locus coeruleus than nucleus basalis and substantia nigra in Alzheimer and parkinson diseases. Arch. Neurol..

[12] Paulus W., Jellinger K. (1991). The neuropathologic basis of different clinical subgroups of Parkinson's disease. J. Neuropathol. Exp. Neurol..

[13] Rinne J.O., Ma S.Y., Lee M.S., Collan Y., Roytta M. (2008). Loss of cholinergic neurons in the pedunculopontine nucleus in Parkinson's disease is related to disability of the patients. Parkinsonism Relat. Disorders.

[14] Cheng Y.C., Su C.H. (2020). Evidence supports PA prescription for Parkinson's Disease: motor symptoms and non-motor features: a scoping review. Int. J. Environ. Res. Publ. Health.

[15] Du S., Long Q., Zhou Y., Fu J., Wu H., Yang L., Liu X. (2026). Transplantation of encapsulated mitochondria alleviates dysfunction in mitochondrial and parkinson's disease models. Cell.

[16] Moon H.E., Paek S.H. (2015). Mitochondrial dysfunction in Parkinson's Disease. Exp. Neurobiol..

[17] Moradi Vastegani S., Nasrolahi A., Ghaderi S., Belali R., Rashno M., Farzaneh M., Khoshnam S.E. (2023). Mitochondrial dysfunction and Parkinson's Disease: pathogenesis and therapeutic strategies. Neurochem. Res..

[18] Jang H.J., Chung K.C. (2022). The ubiquitin-proteasome system and autophagy mutually interact in neurotoxin-induced dopaminergic cell death models of Parkinson's disease. FEBS Lett..

[19] Krashia P., Cordella A., Nobili A., La Barbera L., Federici M., Leuti A., Mercuri N.B. (2019). Blunting neuroinflammation with resolvin D1 prevents early pathology in a rat model of Parkinson's disease. Nat. Commun..

[20] Smajic S., Prada-Medina C.A., Landoulsi Z., Ghelfi J., Delcambre S., Dietrich C., Spielmann M. (2022). Single-cell sequencing of human midbrain reveals glial activation and a Parkinson-specific neuronal state. Brain.

[21] Fan Y., Hu Z., Yan Q.Q., Wan J.J., Liu J. (2025). Whole-exome sequencing and burden analysis identify six novel candidate risk genes and expand the genetic landscape of Parkinson's disease. npj Parkinson's Dis..

[22] Raza C., Anjum R., Shakeel N.U.A. (2019). Parkinson's disease: mechanisms, translational models and management strategies. Life Sci..

[23] Belarbi K., Cuvelier E., Destee A., Gressier B., Chartier-Harlin M.C. (2017). NADPH oxidases in Parkinson's disease: a systematic review. Mol. Neurodegener..

[24] Choi D.H., Cristovao A.C., Guhathakurta S., Lee J., Joh T.H., Beal M.F., Kim Y.S. (2012). NADPH oxidase 1-mediated oxidative stress leads to dopamine neuron death in Parkinson's disease. Antioxid. Redox Signaling.

[25] Cristovao A.C., Choi D.H., Baltazar G., Beal M.F., Kim Y.S. (2009). The role of NADPH oxidase 1-derived reactive oxygen species in paraquat-mediated dopaminergic cell death. Antioxid. Redox Signaling.

[26] Cristovao A.C., Guhathakurta S., Bok E., Je G., Yoo S.D., Choi D.H., Kim Y.S. (2012). NADPH oxidase 1 mediates alpha-synucleinopathy in Parkinson's disease. J. Neurosci..

[27] Fiadeiro M.B., Diogo J.C., Silva A.A., Kim Y.S., Cristovao A.C. (2024). NADPH oxidases in neurodegenerative disorders: mechanisms and therapeutic opportunities. Antioxid. Redox Signaling.

[28] Jiang T., Sun Q., Chen S. (2016). Oxidative stress: a major pathogenesis and potential therapeutic target of antioxidative agents in Parkinson's disease and Alzheimer's disease. Prog. Neurobiol..

[29] Choi D.H., Choi I.A., Lee C.S., Yun J.H., Lee J. (2019). The role of NOX4 in Parkinson's Disease with dementia. Int. J. Mol. Sci..

[30] Kleinschnitz C., Grund H., Wingler K., Armitage M.E., Jones E., Mittal M., Schmidt H.H. (2010). Post-stroke inhibition of induced NADPH oxidase type 4 prevents oxidative stress and neurodegeneration. PLoS Biol..

[31] Ofori K., Ghosh A., Verma D.K., Wheeler D., Cabrera G., Seo J.B., Kim Y.H. (2023). A novel NOX inhibitor alleviates Parkinson's Disease pathology in PFF-Injected mice. Int. J. Mol. Sci..

[32] Boonpraman N., Yoon S., Kim C.Y., Moon J.S., Yi S.S. (2023). NOX4 as a critical effector mediating neuroinflammatory cytokines, myeloperoxidase and osteopontin, specifically in astrocytes in the hippocampus in Parkinson's disease. Redox Biol..

[33] Pourfridoni M., Hakemi Z., Mashayekhi-Sardoo H., Baghcheghi Y. (2026). Molecular pathogenesis of memory impairment in Parkinson's Disease: an exploration of underlying mechanisms. Health Sci. Rep..

[34] Villar-Conde S., Astillero-Lopez V., Gonzalez-Rodriguez M., Villanueva-Anguita P., Saiz-Sanchez D., Martinez-Marcos A., Ubeda-Banon I. (2021). The human hippocampus in Parkinson's Disease: an integrative stereological and proteomic Study. J. Parkinsons Dis..

[35] Liu W., Wang Y., Youdim M.B.H. (2022). A novel neuroprotective cholinesterase-monoamine oxidase inhibitor for treatment of dementia and depression in Parkinson's disease. Ageing and Neurodegenerative Diseases.

[36] Sun C., Armstrong M.J. (2021). Treatment of Parkinson's Disease with cognitive impairment: current approaches and future directions. Behav. Sci..

[37] Erhardt E., Horner A., Shaff N., Wertz C., Nitschke S., Vakhtin A., Ryman S. (2023). Longitudinal hippocampal subfields, CSF biomarkers, and cognition in patients with Parkinson disease. Clin Park Relat Disord.

[38] Surendranathan A., Rowe J.B., O'Brien J.T. (2015). Neuroinflammation in Lewy body dementia. Parkinsonism Relat. Disorders.

[39] Cilia R., Arnaldi D., Ballanger B., Ceravolo R., De Micco R., Del Sole A., van Eimeren T. (2026). Neuroimaging and pathology biomarkers in Parkinson's Disease and parkinsonism. Brain Sci..

[40] Degirmenci Y., Angelopoulou E., Georgakopoulou V.E., Bougea A. (2023). Cognitive impairment in Parkinson's Disease: an updated overview focusing on emerging pharmaceutical treatment approaches. Medicina (Coimbra).

[41] Williams-Gray C.H., Evans J.R., Goris A., Foltynie T., Ban M., Robbins T.W., Barker R.A. (2009). The distinct cognitive syndromes of Parkinson's disease: 5 year follow-up of the CamPaIGN cohort. Brain.

[42] Deng X., Mehta A., Xiao B., Ray Chaudhuri K., Tan E.K., Tan L.C. (2025). Parkinson's disease subtypes: approaches and clinical implications. Parkinsonism Relat. Disorders.

[43] Goldman J.G., Weintraub D. (2015). Advances in the treatment of cognitive impairment in Parkinson's disease. Mov. Disord..

[44] Litvan I., Kieburtz K., Troster A.I., Aarsland D. (2018). Strengths and challenges in conducting clinical trials in Parkinson's disease mild cognitive impairment. Mov. Disord..

[45] Aarsland D., Batzu L., Halliday G.M., Geurtsen G.J., Ballard C., Ray Chaudhuri K., Weintraub D. (2021). Parkinson disease-associated cognitive impairment. Nat. Rev. Dis. Primers.

[46] Goldman J.G., Sieg E. (2020). Cognitive impairment and dementia in parkinson disease. Clin. Geriatr. Med..

[47] Banwinkler M., Theis H., Prange S., van Eimeren T. (2022). Imaging the limbic System in parkinson's Disease-A review of limbic pathology and clinical symptoms. Brain Sci..

[48] Pang C.C., Kiecker C., O'Brien J.T., Noble W., Chang R.C. (2019). Ammon's horn 2 (CA2) of the hippocampus: a long-known Region with a new potential role in neurodegeneration. Neuroscientist.

[49] Phillips O., Ghosh D., Fernandez H.H. (2023). Parkinson disease dementia management: an update of Current evidence and future directions. Curr. Treat. Options Neurol..

[50] Zhang Q., Aldridge G.M., Narayanan N.S., Anderson S.W., Uc E.Y. (2020). Approach to cognitive impairment in Parkinson's Disease. Neurotherapeutics.

[51] Weintraub D., Troster A.I., Marras C., Stebbins G. (2018). Initial cognitive changes in Parkinson's disease. Mov. Disord..

[52] Campos F.L., Carvalho M.M., Cristovao A.C., Je G., Baltazar G., Salgado A.J., Sousa N. (2013). Rodent models of Parkinson's disease: beyond the motor symptomatology. Front. Behav. Neurosci..

[53] Marinus J., Zhu K., Marras C., Aarsland D., van Hilten J.J. (2018). Risk factors for non-motor symptoms in Parkinson's disease. Lancet Neurol..

[54] Muller B., Assmus J., Herlofson K., Larsen J.P., Tysnes O.B. (2013). Importance of motor vs. non-motor symptoms for health-related quality of life in early Parkinson's disease. Parkinsonism Relat. Disorders.

[55] Prakash K.M., Nadkarni N.V., Lye W.K., Yong M.H., Tan E.K. (2016). The impact of non-motor symptoms on the quality of life of Parkinson's disease patients: a longitudinal study. Eur. J. Neurol..

[56] Schapira A.H.V., Chaudhuri K.R., Jenner P. (2017). Non-motor features of Parkinson disease. Nat. Rev. Neurosci..

[57] Bellucci A., Mercuri N.B., Venneri A., Faustini G., Longhena F., Pizzi M., Spano P. (2016). Review: parkinson's disease: from synaptic loss to connectome dysfunction. Neuropathol. Appl. Neurobiol..

[58] Aarsland D., Creese B., Politis M., Chaudhuri K.R., Ffytche D.H., Weintraub D., Ballard C. (2017). Cognitive decline in Parkinson disease. Nat. Rev. Neurol..

[59] Compta Y., Parkkinen L., O'Sullivan S.S., Vandrovcova J., Holton J.L., Collins C., Revesz T. (2011). Lewy- and alzheimer-type pathologies in Parkinson's disease dementia: which is more important?. Brain.

[60] Irwin D.J., White M.T., Toledo J.B., Xie S.X., Robinson J.L., Van Deerlin V., Trojanowski J.Q. (2012). Neuropathologic substrates of Parkinson disease dementia. Ann. Neurol..

[61] Jia X., Wang Z., Yang T., Li Y., Gao S., Wu G., Liang P. (2019). Entorhinal Cortex atrophy in early, drug-naive parkinson's disease with mild cognitive impairment. Aging Dis.

[62] Smith C., Malek N., Grosset K., Cullen B., Gentleman S., Grosset D.G. (2019). Neuropathology of dementia in patients with Parkinson's disease: a systematic review of autopsy studies. J. Neurol. Neurosurg. Psychiatry.

[63] Yu L., Wang T., Wilson R.S., Leurgans S., Schneider J.A., Bennett D.A., Boyle P.A. (2019). Common age-related neuropathologies and yearly variability in cognition. Ann. Clin. Transl. Neurol..

[64] Kehagia A.A., Barker R.A., Robbins T.W. (2013). Cognitive impairment in Parkinson's disease: the dual syndrome hypothesis. Neurodegener. Dis..

[65] Cools R. (2006). Dopaminergic modulation of cognitive function-implications for L-DOPA treatment in Parkinson's disease. Neurosci. Biobehav. Rev..

[66] Schneider J.S., Kortagere S. (2022). Current concepts in treating mild cognitive impairment in Parkinson's disease. Neuropharmacology.

[67] Vaillancourt D.E., Schonfeld D., Kwak Y., Bohnen N.I., Seidler R. (2013). Dopamine overdose hypothesis: evidence and clinical implications. Mov. Disord..

[68] Fang C., Lv L., Mao S., Dong H., Liu B. (2020). Cognition deficits in Parkinson's Disease: mechanisms and treatment. Parkinsons Dis..

[69] Sezgin M., Bilgic B., Tinaz S., Emre M. (2019). Parkinson's disease dementia and lewy body disease. Semin. Neurol..

[70] Buddhala C., Loftin S.K., Kuley B.M., Cairns N.J., Campbell M.C., Perlmutter J.S., Kotzbauer P.T. (2015). Dopaminergic, serotonergic, and noradrenergic deficits in Parkinson disease. Ann. Clin. Transl. Neurol..

[71] van der Zee S., Vermeiren Y., Fransen E., Van Dam D., Aerts T., Gerritsen M.J., De Deyn P.P. (2018). Monoaminergic markers across the cognitive spectrum of lewy body disease. J. Parkinsons Dis..

[72] Ballinger E.C., Ananth M., Talmage D.A., Role L.W. (2016). Basal forebrain cholinergic circuits and signaling in cognition and cognitive decline. Neuron.

[73] Cirrito J.R., Disabato B.M., Restivo J.L., Verges D.K., Goebel W.D., Sathyan A., Sheline Y.I. (2011). Serotonin signaling is associated with lower amyloid-beta levels and plaques in transgenic mice and humans. Proc. Natl. Acad. Sci. U. S. A..

[74] Kotagal V., Spino C., Bohnen N.I., Koeppe R., Albin R.L. (2018). Serotonin, beta-amyloid, and cognition in Parkinson disease. Ann. Neurol..

[75] Olivola E., Pierantozzi M., Imbriani P., Liguori C., Stampanoni Bassi M., Conti M., Stefani A. (2014). Serotonin impairment in CSF of PD patients, without an apparent clinical counterpart. PLoS One.

[76] Politis M., Wu K., Loane C., Turkheimer F.E., Molloy S., Brooks D.J., Piccini P. (2010). Depressive symptoms in PD correlate with higher 5-HTT binding in raphe and limbic structures. Neurology.

[77] Julio F., Ribeiro M.J., Morgadinho A., Sousa M., van Asselen M., Simoes M.R., Januario C. (2022). Cognition, function and awareness of disease impact in early Parkinson's and huntington's disease. Disabil. Rehabil..

[78] Jellinger K.A. (2024). The pathobiology of depression in Huntington's disease: an unresolved puzzle. J. Neural Transm..

[79] Bilal H., Harding I.H., Stout J.C. (2024). The relationship between disease-specific psychosocial stressors and depressive symptoms in Huntington's disease. J. Neurol..

[80] Farzana F., McConville M.J., Renoir T., Li S., Nie S., Tran H., Boughton B.A. (2023). Longitudinal spatial mapping of lipid metabolites reveals pre-symptomatic changes in the hippocampi of Huntington's disease transgenic mice. Neurobiol. Dis..

[81] Fernandez G., Leiva K., Bustos F.J., van Zundert B. (2025). Restoring endogenous Dlg4/PSD95 expression by an artificial transcription factor ameliorates cognitive and motor learning deficits in the R6/2 mouse model of Huntington's disease. Clin. Epigenet..

[82] Jellinger K.A. (2024). Mild cognitive impairment in Huntington's disease: challenges and outlooks. J. Neural Transm..

[83] Anand K.S., Dhikav V. (2012). Hippocampus in health and disease: an overview. Ann. Indian Acad. Neurol..

[84] Das T., Hwang J.J., Poston K.L. (2019). Episodic recognition memory and the hippocampus in Parkinson's disease: a review. Cortex.

[85] Boldrini M., Fulmore C.A., Tartt A.N., Simeon L.R., Pavlova I., Poposka V., Mann J.J. (2018). Human hippocampal neurogenesis persists throughout aging. Cell Stem Cell.

[86] Eriksson P.S., Perfilieva E., Bjork-Eriksson T., Alborn A.M., Nordborg C., Peterson D.A., Gage F.H. (1998). Neurogenesis in the adult human hippocampus. Nat. Med..

[87] Kempermann G., Song H., Gage F.H. (2015). Neurogenesis in the adult hippocampus. Cold Spring Harbor Perspect. Biol..

[88] Spalding K.L., Bergmann O., Alkass K., Bernard S., Salehpour M., Huttner H.B., Frisen J. (2013). Dynamics of hippocampal neurogenesis in adult humans. Cell.

[89] Brown G., Hakun J., Lewis M.M., De Jesus S., Du G., Eslinger P.J., Huang X. (2023). Frontostriatal and limbic contributions to cognitive decline in parkinson's disease. J. Neuroimaging.

[90] Kandiah N., Zainal N.H., Narasimhalu K., Chander R.J., Ng A., Mak E., Tan L.C. (2014). Hippocampal volume and white matter disease in the prediction of dementia in Parkinson's disease. Parkinsonism Relat. Disorders.

[91] Sekiya H., Tsuji A., Hashimoto Y., Takata M., Koga S., Nishida K., Toda T. (2022). Discrepancy between distribution of alpha-synuclein oligomers and Lewy-related pathology in Parkinson's disease. Acta Neuropathol. Commun..

[92] Calabresi P., Castrioto A., Di Filippo M., Picconi B. (2013). New experimental and clinical links between the hippocampus and the dopaminergic system in Parkinson's disease. Lancet Neurol..

[93] Irwin D.J., Grossman M., Weintraub D., Hurtig H.I., Duda J.E., Xie S.X., Trojanowski J.Q. (2017). Neuropathological and genetic correlates of survival and dementia onset in synucleinopathies: a retrospective analysis. Lancet Neurol..

[94] Kalaitzakis M.E., Christian L.M., Moran L.B., Graeber M.B., Pearce R.K., Gentleman S.M. (2009). Dementia and visual hallucinations associated with limbic pathology in Parkinson's disease. Parkinsonism Relat. Disorders.

[95] Hall H., Reyes S., Landeck N., Bye C., Leanza G., Double K., Kirik D. (2014). Hippocampal lewy pathology and cholinergic dysfunction are associated with dementia in parkinson's disease. Brain.

[96] Xu J., Tan S., Zhang X., Zhang M., Xu X. (2026). Hippocampal-striatal interaction in parkinson's disease with mild cognitive impairment. Clin Park Relat Disord.

[97] Zhao F., Behnisch T. (2023). The enigmatic CA2: exploring the understudied Region of the hippocampus and its involvement in Parkinson's disease. Biomedicines.

[98] Novikov N.I., Brazhnik E.S., Kitchigina V.F. (2023). Pathological correlates of cognitive decline in parkinson's disease: from molecules to neural networks. Biochemistry.

[99] Noguchi-Shinohara M., Ono K. (2023). The mechanisms of the roles of alpha-Synuclein, amyloid-beta, and tau protein in the lewy body diseases: pathogenesis, early detection, and therapeutics. Int. J. Mol. Sci..

[100] Lal R., Singh A., Watts S., Chopra K. (2024). Experimental models of parkinson's disease: challenges and opportunities. Eur. J. Pharmacol..

[101] Pingale T., Gupta G.L. (2020). Classic and evolving animal models in parkinson's disease. Pharmacol. Biochem. Behav..

[102] Schneider J.S., Kovelowski C.J. (1990). Chronic exposure to low doses of MPTP. I. Cognitive deficits in motor asymptomatic monkeys. Brain Res..

[103] Klein C., Rasinska J., Empl L., Sparenberg M., Poshtiban A., Hain E.G., Steiner B. (2016). Physical exercise counteracts MPTP-Induced changes in neural precursor cell proliferation in the hippocampus and restores spatial learning but not memory performance in the water maze. Behav. Brain Res..

[104] Hoglinger G.U., Arias-Carrion O., Ipach B., Oertel W.H. (2014). Origin of the dopaminergic innervation of adult neurogenic areas. J. Comp. Neurol..

[105] Dash U.C., Bhol N.K., Swain S.K., Samal R.R., Nayak P.K., Raina V., Jena A.B. (2025). Oxidative stress and inflammation in the pathogenesis of neurological disorders: mechanisms and implications. Acta Pharm. Sin. B.

[106] Tarafdar A., Pula G. (2018). The role of NADPH oxidases and oxidative stress in neurodegenerative disorders. Int. J. Mol. Sci..

[107] Jomova K., Raptova R., Alomar S.Y., Alwasel S.H., Nepovimova E., Kuca K., Valko M. (2023). Reactive oxygen species, toxicity, oxidative stress, and antioxidants: chronic diseases and aging. Arch. Toxicol..

[108] Mankovska I.M., Klymenko O.O., Gonchar O.O., Karasevich N.V., Karaban I.M. (2025). Interplay of oxidative stress and mitochondrial dysfunction in Alzheimer's and Parkinson's diseases: mechanisms and treatment strategies. Neurophysiology.

[109] Olufunmilayo E.O., Gerke-Duncan M.B., Holsinger R.M.D. (2023). Oxidative stress and antioxidants in neurodegenerative disorders. Antioxidants.

[110] Cooke L.E., Rocha E., DiMaio R., Straub A.C., Fazzari M. (2026). The irony of parkinson's disease: converging mechanisms of redox imbalance and ferroptosis. Redox Biol..

[111] Dionisio P.A., Amaral J.D., Rodrigues C.M.P. (2021). Oxidative stress and regulated cell death in parkinson's disease. Ageing Res. Rev..

[112] Flores-Ponce X., Velasco I. (2024). Dopaminergic neuron metabolism: relevance for understanding parkinson's disease. Metabolomics.

[113] Trist B.G., Hare D.J., Double K.L. (2019). Oxidative stress in the aging substantia nigra and the etiology of parkinson's disease. Aging Cell.

[114] Bjorklund G., Peana M., Maes M., Dadar M., Severin B. (2021). The glutathione system in parkinson's disease and its progression. Neurosci. Biobehav. Rev..

[115] Poewe W., Seppi K., Tanner C.M., Halliday G.M., Brundin P., Volkmann J., Lang A.E. (2017). Parkinson disease. Nat. Rev. Dis. Primers.

[116] Zhou Z.D., Yi L.X., Wang D.Q., Lim T.M., Tan E.K. (2023). Role of dopamine in the pathophysiology of parkinson's disease. Transl. Neurodegener..

[117] Chen Y., Qin C., Huang J., Tang X., Liu C., Huang K., Zhou L. (2020). The role of astrocytes in oxidative stress of central nervous system: a mixed blessing. Cell Prolif..

[118] Nayernia Z., Jaquet V., Krause K.H. (2014). New insights on NOX enzymes in the central nervous system. Antioxid. Redox Signaling.

[119] Niu C., Dong M., Niu Y. (2024). Role of glutathione in parkinson's disease pathophysiology and therapeutic potential of polyphenols. Phytother Res..

[120] Tahavvori A., Gargari M.K., Yazdani Y., Mamalo A.S., Beilankouhi E.A.V., Valilo M. (2023). Involvement of antioxidant enzymes in parkinson's disease. Pathol. Res. Pract..

[121] Costa I., Barbosa D.J., Benfeito S., Silva V., Chavarria D., Borges F., Silva R. (2023). Molecular mechanisms of ferroptosis and their involvement in brain diseases. Pharmacol. Ther..

[122] Chakkittukandiyil A., Sajini D.V., Karuppaiah A., Selvaraj D. (2022). The principal molecular mechanisms behind the activation of Keap1/Nrf2/ARE pathway leading to neuroprotective action in parkinson's disease. Neurochem. Int..

[123] Jain R., Vora L., Nathiya D., Khatri D.K. (2025). Nrf2-Keap1 pathway and NLRP3 inflammasome in parkinson's Disease: mechanistic crosstalk and therapeutic implications. Mol. Neurobiol..

[124] Petrillo S., Schirinzi T., Di Lazzaro G., D'Amico J., Colona V.L., Bertini E., Pisani A. (2020). Systemic activation of Nrf2 pathway in Parkinson's disease. Mov. Disord..

[125] Anandhan A., Nguyen N., Syal A., Dreher L.A., Dodson M., Zhang D.D., Madhavan L. (2021). NRF2 loss accentuates parkinsonian pathology and behavioral dysfunction in human alpha-Synuclein overexpressing mice. Aging Dis.

[126] Xu X., Wang Q., Liu Z., Li J., Wang S., Qian L. (2026). Overcoming oxidative stress in parkinson's disease: NADPH oxidase 4 (NOX4) as a potential therapeutic target. Antioxidants.

[127] Alam Z.I., Daniel S.E., Lees A.J., Marsden D.C., Jenner P., Halliwell B. (1997). A generalised increase in protein carbonyls in the brain in Parkinson's but not incidental Lewy body disease. J. Neurochem..

[128] Dexter D.T., Carter C.J., Wells F.R., Javoy-Agid F., Agid Y., Lees A., Marsden C.D. (1989). Basal lipid peroxidation in substantia nigra is increased in Parkinson's disease. J. Neurochem..

[129] Hallqvist J., Toomey C.E., Pinto R., Baldwin T., Doykov I., Wernick A., Heywood W.E. (2025). Multi-omic analysis reveals lipid dysregulation associated with mitochondrial dysfunction in Parkinson'S disease brain. Nat. Commun..

[130] Tresse E., Marturia-Navarro J., Sew W.Q.G., Cisquella-Serra M., Jaberi E., Riera-Ponsati L., Issazadeh-Navikas S. (2023). Mitochondrial DNA damage triggers spread of Parkinson's disease-like pathology. Mol. Psychiatr..

[131] Zhang J., Perry G., Smith M.A., Robertson D., Olson S.J., Graham D.G., Montine T.J. (1999). Parkinson's disease is associated with oxidative damage to cytoplasmic DNA and RNA in substantia nigra neurons. Am. J. Pathol..

[132] Damier P., Hirsch E.C., Zhang P., Agid Y., Javoy-Agid F. (1993). Glutathione peroxidase, glial cells and parkinson's disease. Neuroscience.

[133] Groger A., Kolb R., Schafer R., Klose U. (2014). Dopamine reduction in the substantia nigra of Parkinson's disease patients confirmed by in vivo magnetic resonance spectroscopic imaging. PLoS One.

[134] Sofic E., Lange K.W., Jellinger K., Riederer P. (1992). Reduced and oxidized glutathione in the substantia nigra of patients with Parkinson's disease. Neurosci. Lett..

[135] Bhandari U.R., Danish S.M., Ahmad S., Ikram M., Nadaf A., Hasan N., Ahmad F.J. (2024). New opportunities for antioxidants in amelioration of neurodegenerative diseases. Mech. Ageing Dev..

[136] Yasuhara T., Hara K., Sethi K.D., Morgan J.C., Borlongan C.V. (2007). Increased 8-OHdG levels in the urine, serum, and substantia nigra of hemiparkinsonian rats. Brain Res..

[137] Zeng F., Parker K., Zhan Y., Miller M., Zhu M.Y. (2023). Upregulated DNA damage-linked biomarkers in parkinson's disease model mice. ASN Neuro.

[138] Huin V., Cailliau E., Poupelin C., Garcon G., Dutheil M., Simonin O., group P.s. (2026). Blood-based biomarkers of ferroptosis in parkinson's disease. Neurobiol. Dis..

[139] Smith M.P., Cass W.A. (2007). Oxidative stress and dopamine depletion in an intrastriatal 6-hydroxydopamine model of Parkinson's disease. Neuroscience.

[140] Yoritaka A., Hattori N., Uchida K., Tanaka M., Stadtman E.R., Mizuno Y. (1996). Immunohistochemical detection of 4-hydroxynonenal protein adducts in Parkinson disease. Proc. Natl. Acad. Sci. U. S. A..

[141] Romuk E.B., Szczurek W., Oles M., Gabrysiak A., Skowron M., Nowak P., Birkner E. (2017). The evaluation of the changes in enzymatic antioxidant reserves and lipid peroxidation in chosen parts of the brain in an animal model of parkinson disease. Adv. Clin. Exp. Med..

[142] Chen Y., Luo X., Yin Y., Thomas E.R., Liu K., Wang W., Li X. (2025). The interplay of iron, oxidative stress, and alpha-synuclein in parkinson's disease progression. Mol. Med. (Tokyo).

[143] Deas E., Cremades N., Angelova P.R., Ludtmann M.H., Yao Z., Chen S., Abramov A.Y. (2016). Alpha-synuclein oligomers interact with metal ions to induce oxidative stress and neuronal death in Parkinson's Disease. Antioxid. Redox Signaling.

[144] Ghosh S., Won S.J., Wang J., Fong R., Butler N.J.M., Moss A., Swanson R.A. (2021). alpha-synuclein aggregates induce c-Abl activation and dopaminergic neuronal loss by a feed-forward redox stress mechanism. Prog. Neurobiol..

[145] Liang Y.L., Yang F.G., Tang Q. (2026). The vicious cycle: unraveling the interplay between alpha-synuclein, mitochondrial dysfunction, and neuroinflammation in Parkinson's disease. J. Neurol..

[146] Won S.J., Fong R., Butler N., Sanchez J., Zhang Y., Wong C., Swanson R.A. (2022). Neuronal oxidative stress promotes alpha-Synuclein aggregation in vivo. Antioxidants.

[147] Huang B., Wu S., Wang Z., Ge L., Rizak J.D., Wu J., Li H. (2018). Phosphorylated alpha-Synuclein accumulations and lewy body-like pathology distributed in parkinson's disease-related brain areas of aged rhesus monkeys treated with MPTP. Neuroscience.

[148] Zhang J., Sun B., Yang J., Chen Z., Li Z., Zhang N., Shen L. (2022). Comparison of the effect of rotenone and 1-methyl-4-phenyl-1,2,3,6-tetrahydropyridine on inducing chronic Parkinson's disease in mouse models. Mol. Med. Rep..

[149] Mingo Y.B., Escobar Galvis M.L., Henderson M.X. (2025). alpha-Synuclein pathology and mitochondrial dysfunction: toxic partners in parkinson's disease. Neurobiol. Dis..

[150] Chand J., Fanai H.L., Antony A.S., Subramanian G. (2024). Neurotoxicity by hypoxia an intermediate between alpha-synuclein and mitochondrial dysfunction in parkinson's disease. J. Pharmacol. Pharmacother..

[151] Geibl F.F., Henrich M.T., Xie Z., Zampese E., Ueda J., Tkatch T., Surmeier D.J. (2024). alpha-Synuclein pathology disrupts mitochondrial function in dopaminergic and cholinergic neurons at-risk in Parkinson's disease. Mol. Neurodegener..

[152] Behl T., Madaan P., Sehgal A., Singh S., Anwer M.K., Makeen H.A., Bungau S. (2022). Mechanistic insights expatiating the redox-active-metal-mediated neuronal degeneration in parkinson's Disease. Int. J. Mol. Sci..

[153] Jomova K., Alomar S.Y., Alwasel S.H., Nepovimova E., Kuca K., Valko M. (2024). Several lines of antioxidant defense against oxidative stress: antioxidant enzymes, nanomaterials with multiple enzyme-mimicking activities, and low-molecular-weight antioxidants. Arch. Toxicol..

[154] Zhao Z. (2023). Hydroxyl radical generations form the physiologically relevant Fenton-like reactions. Free Radic. Biol. Med..

[155] Hassan H.A., Ahmed H.S., Hassan D.F. (2024). Free radicals and oxidative stress: mechanisms and therapeutic targets. Hum. Antibodies.

[156] Yao Z., Jiao Q., Du X., Jia F., Chen X., Yan C., Jiang H. (2024). Ferroptosis in Parkinson's disease -- the iron-related degenerative disease. Ageing Res. Rev..

[157] Rizor A., Pajarillo E., Johnson J., Aschner M., Lee E. (2019). Astrocytic oxidative/nitrosative stress contributes to Parkinson's Disease pathogenesis: the dual role of reactive astrocytes. Antioxidants.

[158] Che Y., Zhou Z., Shu Y., Zhai C., Zhu Y., Gong S., Wang J.F. (2015). Chronic unpredictable stress impairs endogenous antioxidant defense in rat brain. Neurosci. Lett..

[159] Camicioli R., Moore M.M., Kinney A., Corbridge E., Glassberg K., Kaye J.A. (2003). Parkinson's disease is associated with hippocampal atrophy. Mov. Disord..

[160] Saleem S., Tabassum S., Ahmed S., Perveen T., Haider S. (2014). Senescence related alteration in hippocampal biogenic amines produces neuropsychological deficits in rats. Pak. J. Pharm. Sci..

[161] Gonzalez-Latapi P., Bayram E., Litvan I., Marras C. (2021). Cognitive impairment in Parkinson's Disease: epidemiology, clinical profile, protective and risk factors. Behav. Sci..

[162] Litvan I., Goldman J.G., Troster A.I., Schmand B.A., Weintraub D., Petersen R.C., Emre M. (2012). Diagnostic criteria for mild cognitive impairment in Parkinson's disease: movement Disorder Society Task Force guidelines. Mov. Disord..

[163] Skorvanek M., Goldman J.G., Jahanshahi M., Marras C., Rektorova I., Schmand B., members of the, M. D. S. R. S. R. C. (2018). Global scales for cognitive screening in Parkinson's disease: critique and recommendations. Mov. Disord..

[164] Ma M.W., Wang J., Zhang Q., Wang R., Dhandapani K.M., Vadlamudi R.K., Brann D.W. (2017). NADPH oxidase in brain injury and neurodegenerative disorders. Mol. Neurodegener..

[165] Boonpraman N., Yi S.S. (2024). NADPH oxidase 4 (NOX4) as a biomarker and therapeutic target in neurodegenerative diseases. Neural Regen. Res..

[166] Lin Z., Ying C., Si X., Xue N., Liu Y., Zheng R., Zhang B. (2025). NOX4 exacerbates Parkinson's disease pathology by promoting neuronal ferroptosis and neuroinflammation. Neural Regen. Res..

[167] Alam Z.I., Jenner A., Daniel S.E., Lees A.J., Cairns N., Marsden C.D., Halliwell B. (1997). Oxidative DNA damage in the parkinsonian brain: an apparent selective increase in 8-hydroxyguanine levels in substantia nigra. J. Neurochem..

[168] Hou L., Sun F., Huang R., Sun W., Zhang D., Wang Q. (2019). Inhibition of NADPH oxidase by apocynin prevents learning and memory deficits in a mouse Parkinson's disease model. Redox Biol..

[169] Wu D.C., Teismann P., Tieu K., Vila M., Jackson-Lewis V., Ischiropoulos H., Przedborski S. (2003). NADPH oxidase mediates oxidative stress in the 1-methyl-4-phenyl-1,2,3,6-tetrahydropyridine model of parkinson's disease. Proc. Natl. Acad. Sci. U. S. A..

[170] Luengo E., Trigo-Alonso P., Fernandez-Mendivil C., Nunez A., Campo M.D., Porrero C., Lopez M.G. (2022). Implication of type 4 NADPH oxidase (NOX4) in tauopathy. Redox Biol..

[171] Bruce-Keller A.J., Gupta S., Knight A.G., Beckett T.L., McMullen J.M., Davis P.R., Keller J.N. (2011). Cognitive impairment in humanized APPxPS1 mice is linked to Abeta(1-42) and NOX activation. Neurobiol. Dis..

[172] Choi D.H., Lee J. (2017). A mini-review of the NADPH oxidases in vascular dementia: correlation with NOXs and risk factors for VaD. Int. J. Mol. Sci..

[173] Han B.H., Zhou M.L., Johnson A.W., Singh I., Liao F., Vellimana A.K., Zipfel G.J. (2015). Contribution of reactive oxygen species to cerebral amyloid angiopathy, vasomotor dysfunction, and microhemorrhage in aged Tg2576 mice. Proc. Natl. Acad. Sci. U. S. A..

[174] Park L., Zhou P., Pitstick R., Capone C., Anrather J., Norris E.H., Iadecola C. (2008). Nox2-derived radicals contribute to neurovascular and behavioral dysfunction in mice overexpressing the amyloid precursor protein. Proc. Natl. Acad. Sci. U. S. A..

[175] Barua S., Kim J.Y., Yenari M.A., Lee J.E. (2019). The role of NOX inhibitors in neurodegenerative diseases. IBRO Rep..

[176] Invernizzi P., Carbone M., Jones D., Levy C., Little N., Wiesel P., study i. (2023). Setanaxib, a first-in-class selective NADPH oxidase 1/4 inhibitor for primary biliary cholangitis: a randomized, placebo-controlled, phase 2 trial. Liver Int..

[177] Jones D., Carbone M., Invernizzi P., Little N., Nevens F., Swain M.G., Levy C. (2023). Impact of setanaxib on quality of life outcomes in primary biliary cholangitis in a phase 2 randomized controlled trial. Hepatol. Commun..

[178] Kaur S., Verma R., Sharma V., Singh T.G. (2025). Targeting NOX inhibitors in neurodegeneration: a therapeutic perspective. Metab. Brain Dis..

[179] Oh E.B., Shin H.J., Yu H., Jang J., Park J.W., Chang T.S. (2024). NADPH oxidase 1/4 dual inhibitor setanaxib suppresses platelet activation and thrombus formation. Life Sci..

[180] Thannickal V.J., Jandeleit-Dahm K., Szyndralewiez C., Torok N.J. (2023). Pre-clinical evidence of a dual NADPH oxidase 1/4 inhibitor (Setanaxib) in liver, kidney and lung fibrosis. J. Cell Mol. Med..

[181] Casas A.I., Geuss E., Kleikers P.W.M., Mencl S., Herrmann A.M., Buendia I., Schmidt H. (2017). NOX4-dependent neuronal autotoxicity and BBB breakdown explain the superior sensitivity of the brain to ischemic damage. Proc. Natl. Acad. Sci. U. S. A..

[182] Heller E., McGurran L., Brown J.K., Love K., Hobbs M., Kim-Han J.S., Han B.H. (2025). Abeta(40) improves cerebrovascular endothelial function via NOX4-Dependent hydrogen peroxide release. Int. J. Mol. Sci..

[183] Zhao Z., Wang R., Ge H., Hou L., Hatano T., Hattori N., Zhao J. (2025). ECHS1-NOX4 interaction suppresses rotenone-induced dopaminergic neurotoxicity through inhibition of mitochondrial ROS production. Free Radic. Biol. Med..

[184] Tao W., Yu L., Shu S., Liu Y., Zhuang Z., Xu S., Zhu X. (2021). miR-204-3p/Nox4 mediates memory deficits in a mouse model of alzheimer's disease. Mol. Ther..

[185] Zhu X., Tao W., Yu L., Jin J., Xu Y. (2020). NOX4 negatively regulates memory functions in APP/PS1 mice. Alzheimer's Dement..

[186] Wang T., An J., Fu X., Sun J., Li H., Han X., Yang W. (2025). GLX351322-Loaded nanoparticles alleviate chronic stress-induced depressive behaviors through inhibition of ferroptosis and oxidative stress. Int. J. Nanomed..

